# Supplementary figures and images for: Novel three-dimensional biochip pulmonary sarcoidosis model
Source: PLoS One. 2021 Feb 4;16(2):e0245805. doi: 10.1371/journal.pone.0245805 (PMC7861546; doi:10.1371/journal.pone.0245805)

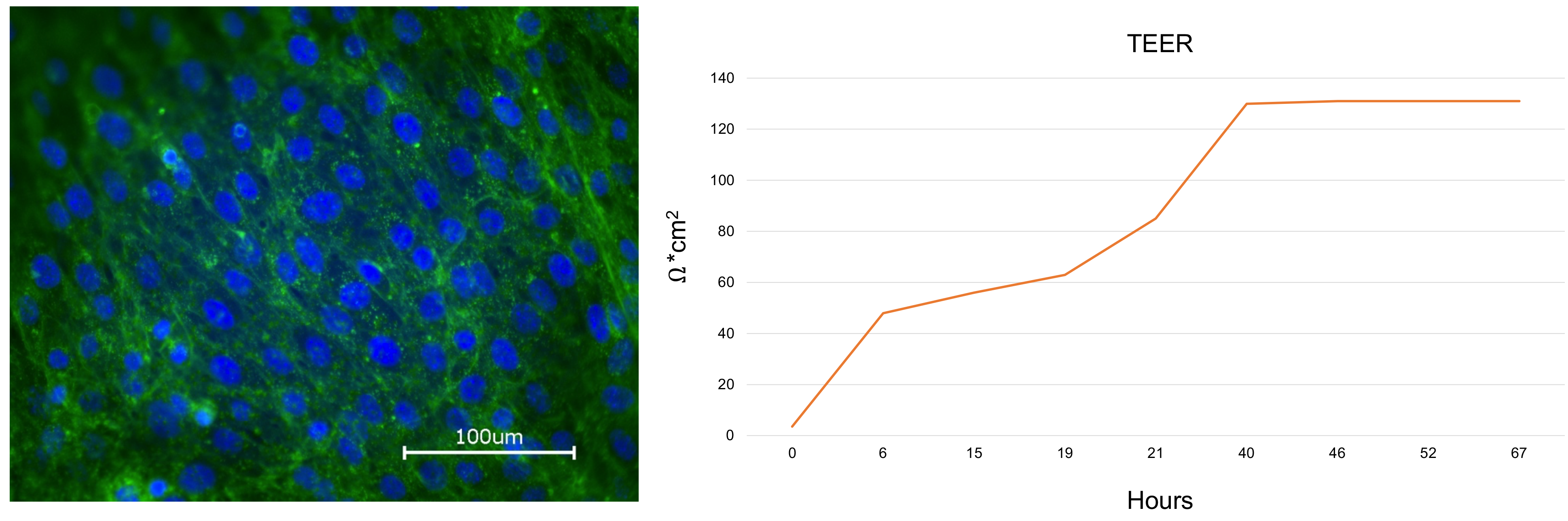

Supplement: S1 Fig — (TIF) [file pone.0245805.s001.tif]
