# Supplementary material for: Novel three-dimensional biochip pulmonary sarcoidosis model
Source: PLoS One. 2021 Feb 4;16(2):e0245805. doi: 10.1371/journal.pone.0245805 (PMC7861546; doi:10.1371/journal.pone.0245805)
Supplement: S1 Data — (DOCX) [file pone.0245805.s002.docx]

Supplemental data

**Novel three-dimensional biochip pulmonary sarcoidosis model**

**Authors and Affiliations**

Tess M Calcagno^1^, Chongxu Zhang^2^, Runxia Tian^3^, Babak Ebrahimi^4^, and Mehdi Mirsaeidi^5^ *

Department of Medicine, University of Miami, Miami, FL, USA^1^

Division of Pulmonary and Critical Care, University of Miami, Miami, FL, USA^2,3,5^

Research and Development, Genix-Engineering, Irvin, California, USA^4^

*Corresponding Author to whom requests for reprints should be addressed

Mehdi Mirsaeidi MD, 1600 NW 10th Ave # 7072B, Miami, Florida, USA 33136

**TEER measurement experiment**

Fibroblasts were cultured and immunofluorescence staining was done to test the functionality of the device as shown in Figure 4. To do so, immunofluorescence (IF) staining was performed by using Wheat Germ Agglutinin (WGA). WGA was bought from Thermo Fisher Scientific (Catalog number: W11261), and manufacturer's instructions were followed. Briefly, fixed cells were washed in PBS buffer 3 times; sufficient amounts of 5.0 μg/mL WGA labeling solution in HBSS buffer were then added to cover cells. The cells were then incubated for 15 minutes at room temperature. When labeling was complete, the labeling solution was removed, and cells were washed three times in HBSS buffer. Mounting Medium with DAPI (Vector lab, Catalog number: H-1200-10) was applied before taking image.


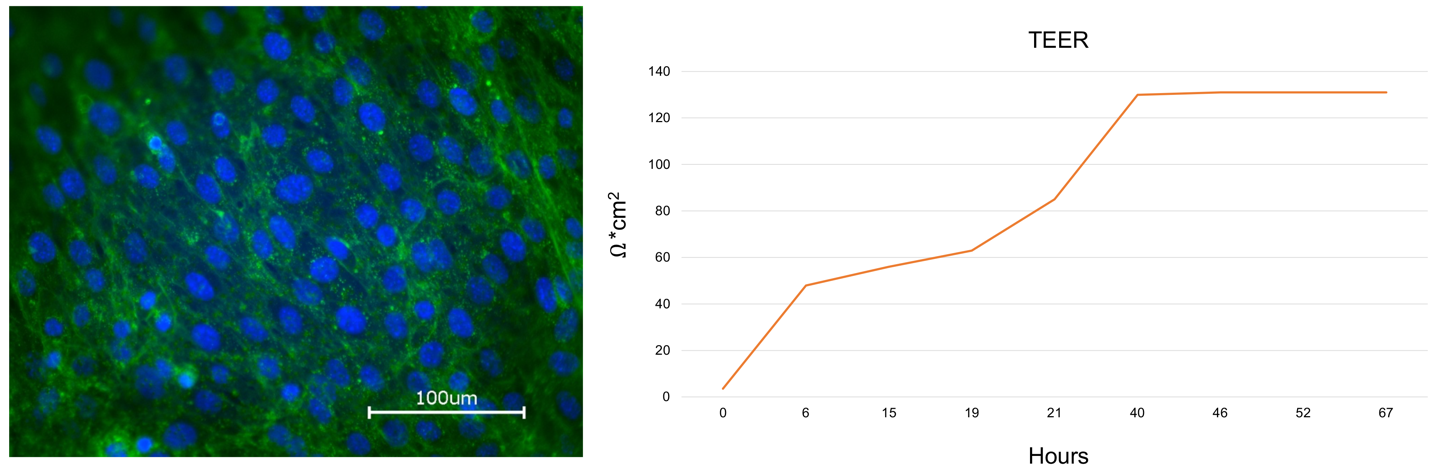


**Figure 4.** Shows immunofluorescence staining of fibroblasts on membrane inside the Lung on chip device with WGA staining. Right image shows TEER results that shows flattening after 40 hours.

**Sarcoidosis-like granuloma in vitro model using peripheral blood mononuclear cells.** We optimized an in vitro sarcoidosis-like granuloma model using PBMC of sarcoidosis patients. We developed an in vitro sarcoidosis-like granuloma model based on culturing PBMC with novel microparticles that are generated from mycobacterial cell walls. PBMC samples were isolated from patients with confirmed pulmonary sarcoidosis and stored frozen inside a -80 °C freezer no longer a year after isolation. PBMC samples were thawed per standard operation and challenged with microparticles from *Mycobacterium abscessus* (MAB) cell walls purified using a protocol from the Bowdish Lab at McMaster University with some modifications. MAB cell wall particles size is less than a micron to 2μm. The microparticles are free of proteins, DNA, and RNA. To prove that the particles are bacteria free, each sample has been cultured and demonstrated no growth before using for the experiment.

The PBMC used in this model were obtained from confirmed treatment-naive sarcoidosis patients, all with a negative IFN-γ release assay (IGRA) for tuberculosis. PBMC were isolated from whole blood samples as previously described, and 5 x 10^6^ cells were cultured in RPMI 1640 medium containing 10% autologous serum in 24-well tissue culture dishes at 37°C in a 5% CO2 atmosphere for 3 days. Microparticles with an equivalent multiplicity of infection (MOI) of 10:1 (and a total endotoxin level of 1.115 EU/ml) were added to the PBMC on the same day they were cultured. On day 3, mature granulomas were present microscopically.

The PBMC in-vitro granuloma model demonstrates a T-helper that is immunophenotyped by cytokine release profile. We explored the cytokine release profile of the granulomas formed in our in vitro model. Supernatants of formed granulomas were collected on Day 3 and analyzed for cytokine concentrations using Illumina multiplex ELISA according to manufacturer recommendations. IL-1β, IL-2R, IL-6, IL-7, IL-8, IL-10, IL-12, IL-15, IFN-𝛼, IFN-*γ, TNF-*𝛼, GM-CSF, CCL2, CCL3, CCL4, CCL5, CXCL9, and CCL11 were measured, and all were found to show significantly higher concentrations in the granuloma samples. Our granuloma model demonstrated a TH1 response with increasing IL-2, IL-7, IL-8, IL-12, IFN-*γ, TNF-*𝛼, and GM-CSF.

**Lung-on-Membrane Model (LOMM)**

Our dual chamber lung model contains normal human bronchial epithelial (NHBE) cells re-differentiated at the air-liquid interface (ALI) on one side and human endothelial cells (Human Lung Microvascular Endothelial Cells, Lonza, Walkersville, MD) on the other side of a transwell^®^ polyester membrane cell culture inserts (12 mm diameter, 0.4 μm pore size; Corning Life Sciences, Amsterdam, The Netherlands). NHBE cells were collected from lungs rejected for transplant at University of Miami where epithelial cells were isolated from upper bronchi and cultured as previously reported. Both sides of the membrane were coated with collagen IV from human placenta (Millipore Sigma, St. Louis, MO, USA). 5 × 10^5^ NHBE cells were cultured on top of the membrane in bronchial epithelial cell growth medium (BEGM) until cells were confluent. The cells were placed on air and fed with ALI Media from bottom chamber thereafter. When NHBE cells were fully differentiated and became ciliated, 2 × 10^5^ endothelial cells were plated on the opposite side of the transwell membrane when membrane was upside down. The upside-down membrane was placed into humidified incubator at 37°C, 5% CO_2_ for 8 h to let endothelial cells to adhere. The transwell was flipped to the original position and both cells lines were feed with a 50:50 mixture of endothelial and epithelia cell media in the bottom chamber and were incubated for 24 h. NHBE cells were washed and the media was changed every 2 days. Two days after adding the endothelial cells, the lung model was used for experiment and the media was changed every 2 days.
