# Supplementary material for: Novel three-dimensional biochip pulmonary sarcoidosis model
Source: PLoS One. 2021 Feb 4;16(2):e0245805. doi: 10.1371/journal.pone.0245805 (PMC7861546; doi:10.1371/journal.pone.0245805)
Supplement: Attachment — Submitted filename: R2 response to Editor.pdf [file pone.0245805.s004.pdf]

UHealth UNIVERSITY OF MIAMI HEALTH SYSTEM
UNIVERSITY OF MIAMI
MILLER SCHOOL of MEDICINE
Miami VA Healthcare System

Dec 8, 2020
Dr. Mária A. Deli, M.D., Ph.D.
Associate Editor
Plos one

Dear editor,

Thank you for your careful review of our manuscript, and especially for the constructive criticism of the reviewers. We are also grateful for the opportunity to revise the manuscript, and respond to their critiques. We believe that we have addressed all concerns and, in doing so, have significantly improved the manuscript. We understand that our manuscript is not perfect, but, has important messages for your readers. A point-by-point response to items raised by the reviewers follows.

Response to Editors.

Response to Reviewer

Reviewer #1: Calcagno et al. provided R1 in a timely fashion. Although several major issues have been addressed, I still do not recommend the publication in this form.
This group has high quality and well written papers, such as PMID 32350353, which shows an example for this paper's aim as for quality. Although PlosOne is a lower impact factor journal than the PMID 323250353, PlosOne is Q1 in its field, therefore high quality of writing and data presentation is expected.

Introduction and Discussion were modified and amended as requested. Still some references are missing, which for example work with Transwell granuloma models, such as PMID 32716613. References 28 and 29 (previously 12 and 13) are still the same, were not corrected, please correct.

Reply: Thanks for suggesting much improved references, they helped to provide more context for our introduction and discussion. A new sentence was added to the discussion as “Walter *et al.* recently developed an in vitro model to study tuberculous mycobacterial granuloma in central nervous system”. We do apologize for the duplication in reference number 29. It has since been removed from the manuscript.

Clarification about the repeat numbers was added, although in the text it is still confusing how many repeats were performed. The Figure legend makes it more understandable.
Reply: A new sentence was added to methods section as “We designed the experiment with 3 replicates, each replicate has 10 samples.” This sentence appears under the subcategory

Cytokine measurements. We also added clarification to the legend of the ELISA results. We hope this adjustment makes the text more understandable.

My two most major concerns are the results section and presentation of the data. Some figures are missing compared to the previous version and the it is not clear why or what was corrected. Still the description of the data is very redundant, although a bit improved compared to the previous version.

Reply:

Thanks for sharing your concerns.

The image about TEER test was removed from the manuscript and added as supplementary figure per the reviewer #2. Reviewer #2 brought up a good point that quality control features of our model (ie TEER testing) were not the central focus of this paper, and belonged in the supplementary data. The data on fibroblast growth to prove functionality of the model was also completely removed as per advice from reviewer #2 to avoid redundancy in results. Instead, cytokine growth measurement is used as our surrogate marker for functionality.

Taking your advice into consideration; we worked to make the results section less redundant.

Results section changes are as follows

1) Paragraph 1: Deletion of sentence two to avoid redundancy. The structure of the granuloma is detailed in the figure legend.
2) Paragraph 2: No changes. The purpose of this paragraph was to outline the progression of our model formation. Development of the macrodevice was the next essential step.
3) Paragraph 3: No changes. This paragraph outlines the development of the bilayered lung model with its various components.
4) Paragraph 4: Sentence 2 deleted to avoid redundancy. Sentence 2 was essentially a descriptor of how the BOSGM appears; we agree this was redundant in the setting of the descriptor of appearance in legend of figure 4. We added in a sentence to clarify what the BOSGM is comprised of. We hope this will make it easier for the reader to understand the full picture.
5) Paragraph 5: We clarified the purpose of cytokine testing. Cytokine expression to show the functionality of the model.

Overall, we explain our results in an improved and structured fashion → Granuloma formation, macrodevice development → Bilayer lung model development → BOSGM final product → Cytokine test against control. Figure legends do a nice job of describing the visuals without *in text* redundancy.

Also the answers for the reviewers' questions were extremely redundant and minimalistic, which is very unprecedented and unacceptable. Several questions of the previous revisions were not answered or are not found well in the text. Marked-up copy should have been provided to help the work of the reviewers.

Reply: We hope that in the round of edits, we provide a more complete view of the changes made and provide more adequate responses. We agree that highlighted version would help you to find extensive changes in the manuscript. We added highlighted **Revised 1** for your review. We hope we have adequately addressed any pending concerns in this round of edits.

Thank you again for taking the time to help us publish our message and share our research with readers of PlosOne.

All the best,

Mehdi Mirsaeidi, MD, MPH
Division of Pulmonary, Critical Care,
Sleep and Allergy
Director of Sarcoidosis Program
Miami VA Medical Center
Department of Medicine
University of Miami, Miller School of Medicine
1600 NW 10th Ave # 7072A
Miami, FL 33136
(305) 243-9227
Email: msm249@miami.edu
