# Supplementary material for: Novel three-dimensional biochip pulmonary sarcoidosis model
Source: PLoS One. 2021 Feb 4;16(2):e0245805. doi: 10.1371/journal.pone.0245805 (PMC7861546; doi:10.1371/journal.pone.0245805)
Supplement: Attachment — Submitted filename: R1 response to Editor .docx [file pone.0245805.s003.docx]

October 23, 2020

Dr. Mária A. Deli, M.D., Ph.D.

Associate Editor
Plos one

Dear editor,

Thank you for your careful review of our manuscript, and especially for the constructive criticism of the reviewers. We are also grateful for the opportunity to revise the manuscript, and respond to their critiques. We believe that we have addressed all concerns and, in doing so, have significantly improved the manuscript. We understand that our manuscript is not perfect, but, has important messages for your readers. A point-by-point response to items raised by the reviewers follows.

Response to Editors.

**Response to Reviewer**

Reviewer #1:

Cacagno et al. developed an in vitro sarcoidosis granuloma model derived from the blood of patients. These 3D cellular structures were then added to a novel microfluidics system using regular Transwell system to model air liquid interface (ALI), and cell-cell interactions, cell aggregate-cell interactions and cytokine release was characterized. Generally the paper contains very interesting material related to the field, since no good lung granuloma-lung tissue (or other barrier tissue) in vitro model exists.

Although non-referenced, but important papers in this research area should be cited such as (PMIDs): 28174307; 32974300; 32716613.

Reply: Thanks for reference! It has been added.

The major problems with the paper are not the findings, but with the presentation of the data.

Reply: Thanks for comment. We restructured the manuscript.

 Introduction is too general, contains repetitions and the references are not always matching the text in a required fashion. Authors claim that neither human, animal or in vitro models are good to model the disease, which is important when one wants to state the limitations of different experimental systems. Here biochip models are claimed as too low throughput and cell culture models as redundant. Still authors try to present a new microfluidic-ALI-cell culture granuloma-model. Although one understands the model, the reasoning behind is not very logical.

Reply: Thanks for comment. We manuscript has been revised per your comment.

Methods section does not contain any ethical statements, although cells were isolated from patients. This has to be added.

Reply: Thanks for comment. Ethical statement was added.

Discussion contains some methodology about the device, which should be inserted here. 10 paralells mean less than 4 repeats with the system / group?

Reply: Thanks for comment. (N) is 10 and has been corrected.

 Results section is extremely redundant. Results have to be presented more in detail, not just as one would describe a figure legend.

Reply: Thanks for comment and apology for mistakes. The discussion has been rewritten.

Please also double-check all references, for which I also indicated some ideas: - Ref 2, 3: the text is matching the logic of the paper, but the references are not very well chosen, they are too general.

Reply: Thanks for comment. The reference section has been corrected

To discuss the limitations of animal models, special sarcoidosis animal models should be mentioned and discussed.

Reply: Thanks for comment. It has been added.

Ref. 3. Is a German language paper matching the content of the text, but is also too general and is not available to read to wider audience. - Ref 4-7. - About biochip modeling and its versatility. These references are coming from 2 groups, where 2 papers is citing heart tissue monitoring. Plenty of papers are to be found about lung models, and in other tissue barrier models, please use a more broad range of citations when referring to this very wide field. - Sentence before Ref. 9. is almost the same as sentence before ref.2., so it is a repetition, please remove. - Refs 12 and 13 are the same, please correct.

Reply: Thanks for comment. We have enriched the reference section.

 In general the paper contains good data, and the biochip system looks valuable, but the manuscript has to be re-formatted, carefully checked and some parts re-written. Also the title (and the text) should be re-formatted: the word "organoid" suggests a stem-cell based approach, which is not the case here.

Reply: Thanks for comment. The paper has been improved with your comments and title has been changed.

**Reviewer #2:**

They further challenged the air-lung interface of the organoid sarcoidosis model with microparticles to demonstrate the functionality of the device for experimental studies.  Overall, the manuscript seems to be prepared in haste without sufficient detail for the readers to follow the narrative resulting in major deficiencies and, therefore, is not recommended for publication in its current form. The field of lung-on-a-chip has rapidly been developing in the last decade and is reaching maturity to a certain extent. Introducing granuloma formation to mimic in vitro sarcoidosis in the lungs is certainly a nice contribution. However, the text is rather confusing as to the origin of granuloma formation; is it from PBMC taken from blood of patients with sarcoidosis or is it induced by in-house developed microparticles?

Reply: Thanks for comment. We added more details on the development of in vitro granuloma.

The images in Figure 1 seem to be taken from a culture dish; how these cultures are utilized for the development of 3-D OSGM? No protocol has been detailed.

Reply: Thanks for comment. The method section has been rewritten to add the methodology of 3D BOSG.

 The 3 chambers in the system design shown in Figure 2 is an artefact as all chambers are identical; it is rather easy to design a similar device with 6 chambers or assemble two devices with the same number of devices. The authors should completely mute this aspect of the device.

Reply: We agree with your great point. Your comment has been applied.

 How the cross-section in Figure 3 was taken; presumably this is taken from the previously developed dual-chambered lung-on-membrane model which is not new? The endothelial layer seems to be rather sparse not quite fully confluent; is this an important issue, Can this be a reliable model?

Reply: Thanks for comment. This image is from our published model. Endothelial cells are so thin and easily wall off when the paraffin block gets cut.

 What is the role of the fibroblast cells shown in Figure 4; the lung model has already been developed and includes only epithelial and endothelial cells. The fibroblast discussion is completely superficial and should be removed although the image is nice. A similar image for the endothelial cells would be much more desired.

Reply: Thanks for comment. We moved fibroblasts experiments to supplemental file.

Thank you

Mehdi Mirsaeidi, MD, MPH

Division of Pulmonary, Critical Care,

Sleep and Allergy

Director of Sarcoidosis Program

Miami VA Medical Center

Department of Medicine

University of Miami, Miller School of Medicine

1600 NW 10th Ave # 7072A

Miami, FL 33136

(305) 243-9227

Email: msm249@miami.edu
